# The Core-Shell Heterostructure CNT@Li_2_FeSiO_4_@C as a Highly Stable Cathode Material for Lithium-Ion Batteries

**DOI:** 10.1186/s11671-019-3165-x

**Published:** 2019-10-17

**Authors:** Tao Peng, Wei Guo, Yingge Zhang, Yangbo Wang, Kejia Zhu, Yan Guo, Yinghui Wang, Yang Lu, Hailong Yan

**Affiliations:** 1 School of Physics and Electronic Engineering, Xinyang Normal University, Xinyang, 464000 People’s Republic of China; 2 Key Laboratory of Microelectronics and Energy of Henan Province, Henan Joint International Research Laboratory of New Energy Storage Technology, Xinyang Normal University, Xinyang, 464000 People’s Republic of China

**Keywords:** Lithium-ion batteries, Cathode, Li_2_FeSiO_4_, Core-shell structure

## Abstract

The reasonable design of nanostructure is the key to solving the inherent defects and realizing a high performance of Li_2_FeSiO_4_ cathode materials. In this work, a novel heterostructure CNT@Li_2_FeSiO_4_@C has been designed and synthesized and used as a cathode material for lithium-ion battery. It is revealed that the product has a uniform core-shell structure, and the thickness of the Li_2_FeSiO_4_ layer and the outer carbon layer is about 19 nm and 2 nm, respectively. The rational design effectively accelerates the diffusion of lithium ions, improves the electric conductivity, and relieves the volume change during the charging/discharging process. With the advantages of its specific structure, CNT@Li_2_FeSiO_4_@C has successfully overcome the inherent shortcomings of Li_2_FeSiO_4_ and shown good reversible capacity and cycle properties.

## Introduction

Lithium-ion batteries (LIBs) have been widely used in portable electronic devices and electric vehicles because of the advantages of high-efficiency energy conversion, long cycling life, high energy density, and low self-discharge [1–3]. However, new generation LIBs with higher energy density, higher rate capability, and higher cycling performance are urgently needed to meet the development of electric vehicles [4–6]. It has been recognized that exploiting new cathode materials is of great significance for the development of the new generation LIBs, as the performance of LIBs is closely related to the properties of cathode materials. In the last few years, the Li_2_FeSiO_4_ cathode material has aroused great attention due to its inexpensive, good chemical stability and environmentally friendly. Especially, it is potential to insert/extract two lithium ions for each molecular unit having a capacity of 332 mAh g^−1^ in theory [7, 8]. Yet it is hard to realize the two Li storages in practical applications, because of the low conductivity (~ 6 × 10^− 14^ S cm^−1^) and the lithium-ion diffusion coefficient (~ 10^− 14^ cm^2^ s^−1^) of Li_2_FeSiO_4_ [9–12]. Hence, it is very important to improve the electronic conductivity and lithium-ion transport ability of Li_2_FeSiO_4_ cathode to optimize the performance of LIBs. Many efforts have been made to solve these problems, such as synthesizing nanoscale Li_2_FeSiO_4_ [13–15] and coating the surface with a carbon material [16–18]. However, it often encounters two problems during the synthesis process. One problem is that it is difficult to synthesize a pure-phase Li_2_FeSiO_4_ product, and some accompanying impurities such as Fe_3_O_4_ or Li_2_SiO_3_ are often unavoidable, which is adverse to the practice capacity [19, 20]. The other problem is that the nanostructure of Li_2_FeSiO_4_ often encounters damage during the annealing treatment.

In this study, a core-shell heterostructure CNT@Li_2_FeSiO_4_@C cathode material has been synthesized. Compared with the reported work about Li_2_FeSiO_4_ cathode material, the CNT@Li_2_FeSiO_4_@C material exhibits the advantage of no impurity phase and structural integrity by adjusting the chemical reagent metering ratio and reaction conditions. In addition, the inner layer of CNT can increase conductivity. The thickness of the Li_2_FeSiO_4_ in the middle layer is only 20–25 nm, which benefits the Li^+^ diffusion. And the outer amorphous carbon layer can improve conductivity and protect the internal frame structure. Therefore, the core-shell heterostructure CNT@Li_2_FeSiO_4_@C exhibits a significant improvement in specific capacity, cycle stability, and rate performance.

## Methods

### Materials and Synthesis

The CNT@Li_2_FeSiO_4_@C was fabricated by a step-by-step preparation process. First, the CNT@SiO_2_ coaxial structure was prepared by a sol-gel coating process [21, 22]. Typically, 8 mg of CNTs was dispersed in the mixture of 80 mL of ethanol and 30 mL of deionized water, and then the mixture was sonicated for 30 min to form a homogeneous solution. Then, 2 mL of NH_3_·H_2_O (25–28 wt.%) and 0.16 g of cetyltrimethylammonium bromide (CTAB) were added into the above solution under sonicated and kept for 20 min. The mixed solution containing 0.45 mL of tetraethoxysilane (TEOS) and 40 mL of ethanol was dripped into the above solution under magnetic stirring for more than 40 min, which was followed by stirring for 10 h. The CNT@SiO_2_ product was obtained by centrifugation and cleaning with deionized water and ethanol. Next, the CNT@Li_2_FeSiO_4_ is obtained by solid-phase sintering. The aqueous solution of 0.334 g LiAc·2H_2_O and 0.734 g Fe(NO_3_)_3_·9H_2_O was successively dissolved in 30 mL ethanol under stirring for 20 min. The obtained CNT@SiO_2_ was added to the solution and sonicated for 40 min and magnetic stirring for 20 min. Then, it was transferred to a vacuum dryer of 80 °C for 12 h. The obtained dry powder was first calcined at 400 °C for 2 h and then heated at 650 °C for 10 h in the argon atmosphere; as a result, the CNT@Li_2_FeSiO_4_ was prepared. The CNT@Li_2_FeSiO_4_@C was prepared by using glucose as a carbon source. 0.2 g of CNT@Li_2_FeSiO_4_ was dispersed by ultrasonic for 40 min in 40 mL ethanol. Then, the solution containing 0.1 g of glucose was added to the solution. The resulting solution was placed in a vacuum dryer at 80 °C for 6 h. Then, the product was calcined 400 °C for 4 h in argon atmosphere to get the CNT@Li_2_FeSiO_4_@C cathode material.

### Materials Characterization

The crystalline structure of the CNT@Li_2_FeSiO_4_@C and CNT@Li_2_FeSiO_4_ was characterized by X-ray diffraction (XRD, D2 PHASER Bruker) in the 2θ ranging from 10° to 80° with Cu-Kα radiation (*λ* = 1.5418 Å) radiation at 30 kV and 10 mA. The chemical elements of the materials were verified by X-ray photoelectron spectroscopy (XPS, K-ALPHA 0.5EV) system. The scanning electron microscopy (FESEM, S-4800) and transmission electron microscopy (TEM, Tecnai G2 F 20) were used to observe the structure and morphology of the materials. The distribution of element in the composite was revealed by energy-dispersive X-ray spectroscopy (EDX). The carbon content of the composite was examined by thermogravimetric analyzer (TGA) curve (STD Q600 TA) with a speed of 10 °C min^−1^ from RT to 800 °C.

### Electrochemical Measurements

The cathode was made by 10 wt.% polyvinylidene fluoride (PVDF), 20 wt.% acetylene black, and 70 wt.% active material dispersed in *N*-methyl-2-pyrrolidone (NMP) to form a consistent slurry. Then the slurry was coated onto the Al foil and dried under vacuum at 80 °C for more than 12 h. The mass loading of the active material for CNT@Li_2_FeSiO_4_@C and CNT@Li_2_FeSiO_4_ electrode was about 1.5 mg/cm^2^. At last, the half cells were assembled in an argon-filled glove box. The battery was tested for charge and discharge using a Neware battery system over a voltage window of 1.5~4.8 V. Cyclic voltammetric (CV) measurement was carried out on a VMP3 multichannel electrochemical workstation (France, Bio-logic) at different scan rates.

## Results and Discussion

**Fig. 1:**
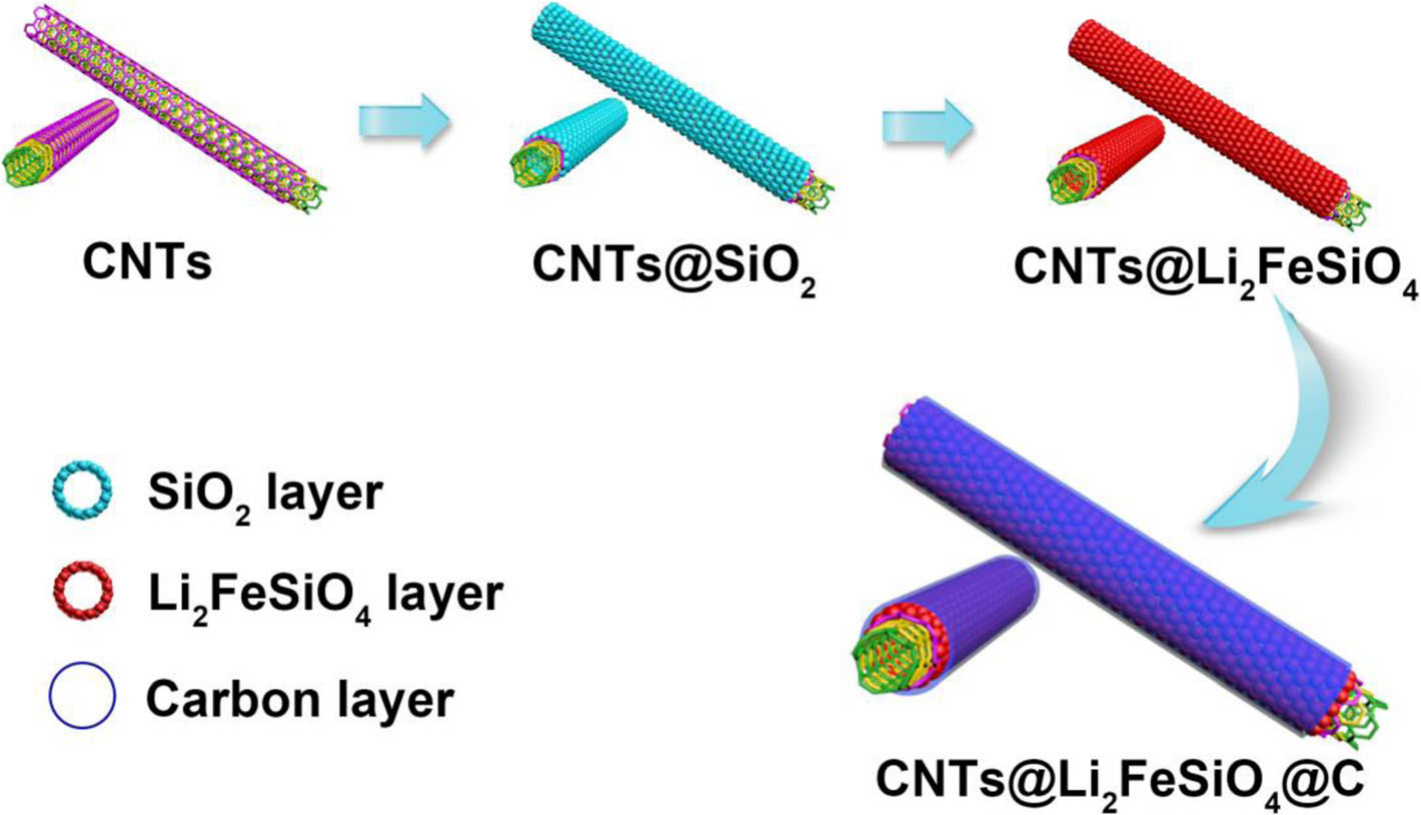
Schematic diagram of synthesis of the core-shell heterostructure CNT@Li_2_FeSiO_4_@C

The schematic diagram of the preparation process of the CNT@Li_2_FeSiO_4_@C material is shown in Fig. 1. First, the CNT@SiO_2_ was prepared by a simple sol-gel method. By controlling the dosage of CNT and TEOS, the purpose of accurately controlling the thickness of the Li_2_FeSiO_4_ layer was achieved. Second, CNT@Li_2_FeSiO_4_ with good crystallinity was obtained by annealing at 650 °C for 10 h in argon atmosphere. Finally, the CNT@Li_2_FeSiO_4_ was coated with amorphous carbon only at 400 °C to achieve the core-shell heterostructure CNT@Li_2_FeSiO_4_@C.

**Fig. 2:**
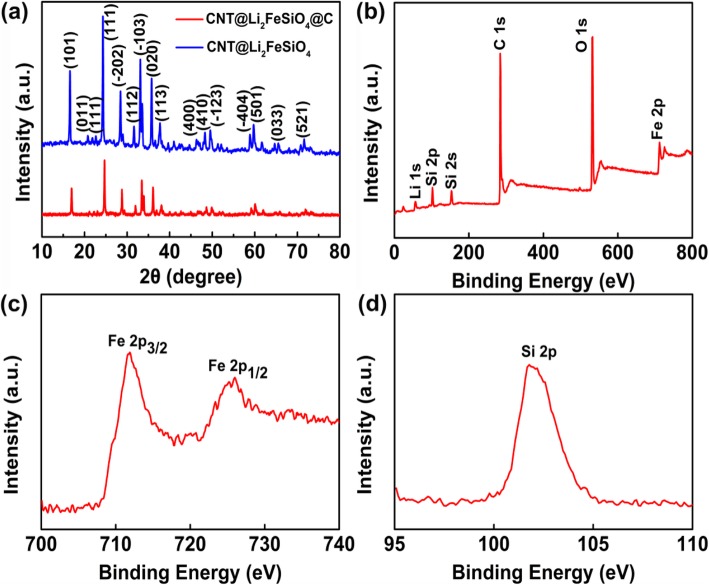
**a** XRD of CNT@Li_2_FeSiO_4_ and CNT@Li_2_FeSiO_4_, **b** XPS full spectra of CNT@Li_2_FeSiO_4_@C, and high-resolution spectra of **c** Fe 2p and **d** Si 2p

Figure 2a depicts the XRD patterns of CNT@Li_2_FeSiO_4_ and CNT@Li_2_FeSiO_4_@C**.** The diffraction peaks of Li_2_FeSiO_4_ are consistent with the monoclinic structure with p2_1_/n space group. Moreover, no XRD diffraction peak of impurities (such as Fe_3_O_4_ and Li_2_SiO_3_) is observed, which confirms the purity of the obtained product. The main element and the surface valence state of CNT@Li_2_FeSiO_4_@C are studied by XPS (Fig. 2b–d). Figure 2b shows the full spectrum of Li_2_FeSiO_4_, including Li 1s, Si 2p, Si 2s, C 1s, O 1s, and Fe 2p. Figure 2c shows the Fe2p spectrum, and two peaks at 710.3 eV and 723.8 eV are attributed to Fe 2p_3/2_ and Fe 2p_1/2_ of Fe^2+^, respectively. The binding energy satellite peak at 710.4 eV is 4.6 eV lower than the binding energy satellite peak at 715.0 eV, which further indicates that only Fe^2+^ exists in Li_2_FeSiO_**4**_ nanocrystals [23]. Figure 2d shows that the peak at Si 2p at 101.8 eV is substantially consistent with the peak at Si^4+^ in polysiloxane, which proves the formation of orthogonal silicate structure [SiO_4_] [24, 25].

**Fig. 3:**
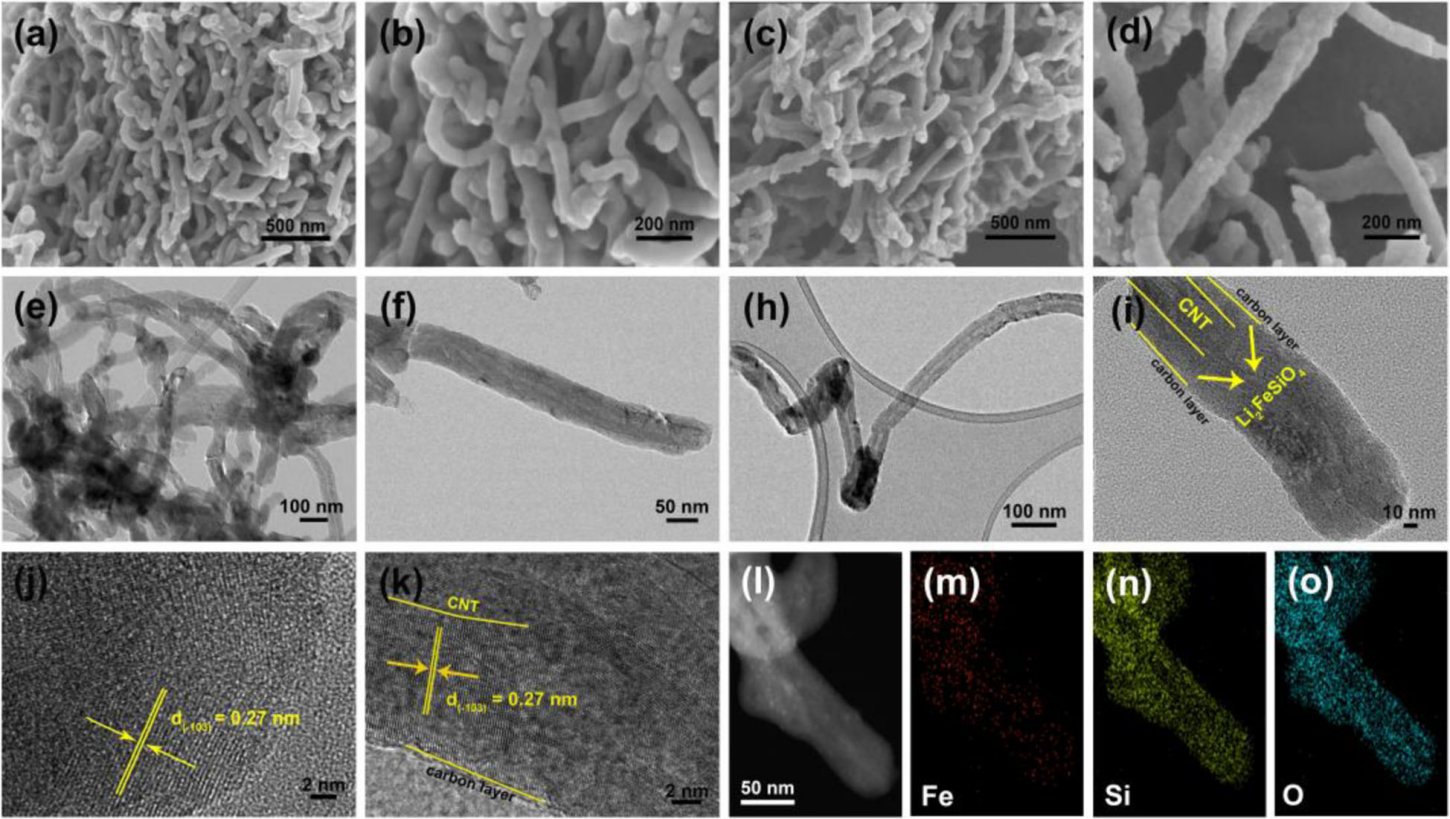
**a**, **b** SEM images of CNT@Li_2_FeSiO_4_, **c** and **d** SEM images of CNT@Li_2_FeSiO_4_@C. **e** and **f** TEM images of CNT@Li_2_FeSiO_4_, **h** and **i** TEM images of CNT@Li_2_FeSiO_4_@C; **j** and **k** HRTEM of CNT@Li_2_FeSiO_4_@C and CNT@Li_2_FeSiO_4_@C, respectively; **l**–**o** EDX elemental mappings of Fe, Si, and O

Scanning electron microscopy (SEM) reveals the morphology and structure of CNT (Additional file 1: Figure S1A), CNT@SiO_2_ (Additional file 1: Figure S1B), CNT@Li_2_FeSiO_4_ (Fig. 3a, b), and CNT@Li_2_FeSiO_4_@C (Fig. 3c, d). It is worth noting that the one-dimensional nanostructure of both CNT@Li_2_FeSiO_4_ and CNT@Li_2_FeSiO_4_@C is kept and no free Li_2_FeSiO_4_ particles are observed. The TG result confirms that the carbon content of CNT@Li_2_FeSiO_4_ and CNT@Li_2_FeSiO_4_@C was 16.93% and 22.69%, respectively (Additional file 1: Figure S2).

The structure of CNT@Li_2_FeSiO_4_ and CNT@Li_2_FeSiO_4_@C is further confirmed by transmission electron microscopy (TEM). The core-shell structure of CNT@Li_2_FeSiO_4_ can be visually discerned from Fig. 3e and f. The total diameter of CNT@Li_2_FeSiO_4_ is about 70 nm and the thickness of the outer layer Li_2_FeSiO_4_ is 20 nm. The core-shell heterostructure of CNT@Li_2_FeSiO_4_@C can be clearly confirmed in Fig. 3h and i. Moreover, Fig. 3i further shows the one-dimensional nanostructure of the CNT@Li_2_FeSiO_4_@C with a diameter of about 85 nm. The inner layer of CNT has a diameter of about 40 nm, and the middle layer of Li_2_FeSiO_4_ has a thickness of about 16–22 nm. And the thickness of the outer carbon layer is around 2 nm. Figure 3j and k shows the HR-TEM of CNT@Li_2_FeSiO_4_ and CNT@Li2FeSiO4@C, respectively. The lattice fringe with a distance of 0.27 nm coincides with the (− 103) lattice spacing of the orthorhombic Li_2_FeSiO_4_. Figure 3k also shows the thickness of the outer amorphous carbon is around 1.5 nm. Figure 3l–o are elemental mapping analysis of CNT@Li_2_FeSiO_4_, which demonstrates the coexistence and uniform distribution of Fe, Si, and O elements.

**Fig. 4:**
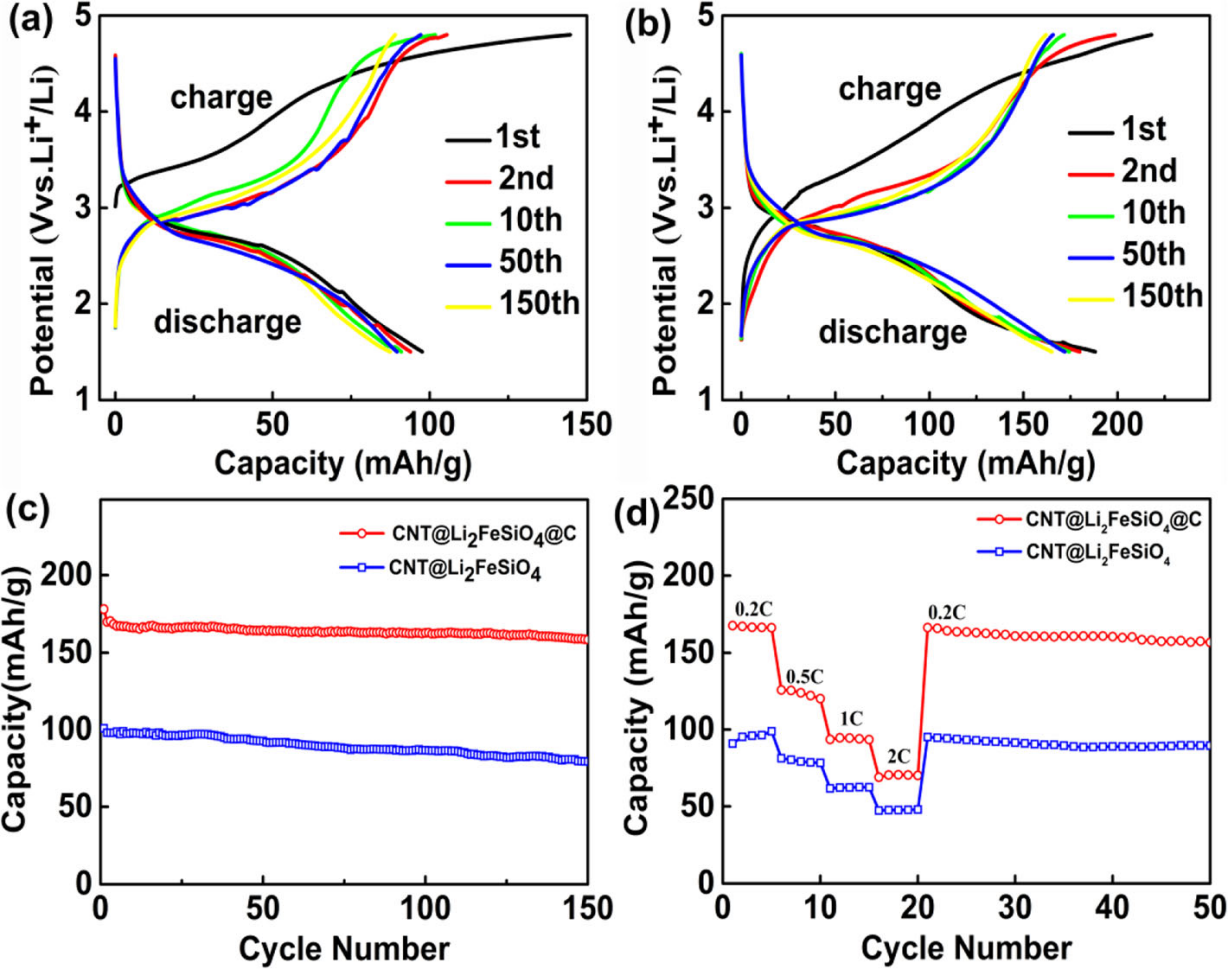
**a** The charge/discharge curves of CNT@Li_2_FeSiO_4_ electrode at the rate of 0.2 C, **b** the charge/discharge curves of CNT@Li_2_FeSiO_4_@C electrode at the rate of 0.2 C, **c** the cycling performance of CNT@Li_2_FeSiO_4_ and CNT@Li_2_FeSiO_4_@C electrode, and **d** the rate performance of CNT@ Li_2_FeSiO_4_ and CNT@ Li_2_FeSiO_4_@C electrode

The cycling performance of CNT@Li_2_FeSiO_4_ and CNT@Li_2_FeSiO_4_@C was tested on a Neware battery test system with a voltage window of 1.5–4.8 V at a current density of 0.2 C. As shown in Fig. 4a, the first discharge specific capacity of the CNT@Li_2_FeSiO_4_ is 100.8 mAh g^−1^ and the capacity retention after 2, 10, 50, and 150 cycles was 95.2%, 92.8%, 91%, and 78.2%, respectively. By contrast, the CNT@Li_2_FeSiO_4_@C has the charge capacity of 207 mAh g^−1^ and the discharge capacity of 178 mAh g^−1^ in the initial cycle (Fig. 4b). After 2, 10, 50, and 150 cycles, the discharge capacity retention of the CNT@Li_2_FeSiO_4_ @C electrode are remained at 95.5%, 93.3%, 92.4%, and 89.3%, respectively. Obviously, CNT@Li_2_FeSiO_4_@C has much higher capacity and better cycling performance compared to CNT@Li_2_FeSiO_4_, which should be due to its much higher conductivity [26, 27]. As far as we know, the cycling characteristics of CNT@Li_2_FeSiO_4_@C in this work exhibits much better cycling performance compared with that of previous reports. For instance, porous Li_2_FeSiO_4_/C nanocomposite prepared by tartaric acid-assisted sol-gel method had an initial discharge capacity of 176.8 mAh g^−1^ at 0.5 C and a reversible capacity of 132.1 mAh g^−1^ at 1 C after 50 cycles [28]. Reduced graphene oxide modified Li_2_FeSiO_4_/C composite was synthesized by a citric-acid-based sol-gel method that could deliver a reversible capacity of 178 mAh g^−1^ at 0.1 C and a capacity retention of 94.5% after 40 cycles [29]. The reason for the better electrochemical properties can be summarized as the following three parts. First, the combination of CNT and Li_2_FeSiO_4_ can improve the electrical conductivity of the material. Second, Li_2_FeSiO_4_ with a thickness of only 20–25 nm benefits the diffusion of lithium ion. Last, the carbon layer coated on the outside of the Li_2_FeSiO_4_ can protect the inner structure and further improve the conductivity. In addition, the cycling performance plots of CNT@Li_2_FeSiO_4_ and CNT@Li_2_FeSiO_4_@C in Fig. 4c further validate the above statement. It can be seen that the CNT@Li_2_FeSiO_4_@C has higher cycle performance and capacity than that of CNT@Li_2_FeSiO_4_ at a current density of 0.2 C. The rate performance of CNT@Li_2_FeSiO_4_ and CNT@Li_2_FeSiO_4_@C is shown in Fig. 4d. It can be observed that the discharge capacity of CNT@Li_2_FeSiO_4_ is 98.8 mAh g^−1^, 81.3 mAh g^−1^, 78.6 mAh g^−1^, and 62.4 mAh g^−1^ at 0.2, 0.5, 1, and 2 C, respectively. While CNT@Li_2_FeSiO_4_@C cathode exhibits a much better rate performance, the discharge capacity of 167.7 mAh g^−1^, 125.8 mAh g^−1^, 94.6 mAh g^−1^, and 70.6 mAh g^−1^ is achieved at 0.2, 0.5, 1, and 2 C, respectively. These performances are better than those described in other similar reports [30–32].

**Fig. 5:**
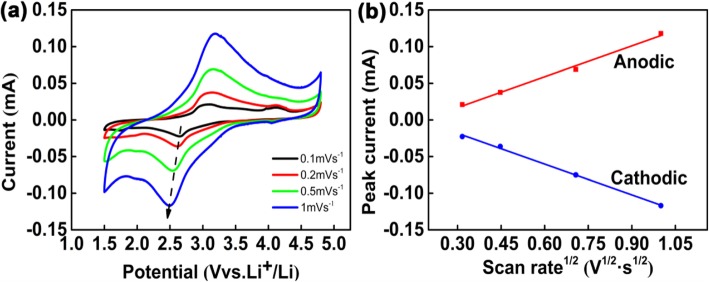
Kinetic analysis of CNT@Li_2_FeSiO_4_@C using CV. **a** CV profiles at various scan rates. **b** peak current as a function of square root of scan rates

The CV curves are further applied to analyze the kinetic characteristic of CNT@Li_2_FeSiO_4_@C cathode material. Figure 5a shows a CV profile of CNT@Li_2_FeSiO_4_@C at different scan rates from 0.1 to 1.0 mV s^−1^.

The main redox couple potentials caused by the Fe^2+^/Fe^3+^ reaction are 3.1 V (anodic) and 2.7 V (cathodic), respectively, which is in accordance with the galvanostatic platform curve. It is worth noting that only one main redox couple potentials at 3.1 V (anodic) and 2.7 V (cathodic) corresponding to the Fe^2+^/Fe^3+^ reaction are observed, and Fe^3+^/Fe^4+^ redox couple is confirmed to be inexistent. And the CV result is in accordance with the dQ/dV vs. voltage data (Additional file 1: Figure S3). So we can conclude that only one Li^+^ per formula unit is inserted and extracted during the charge/discharge process.

In addition, the peak position of the redox peak changes a little with the increasing of scanning rate, indicating the small polarization reaction of CNT@Li_2_FeSiO_4_@C cathode material [28]. The diffusion coefficient of lithium ions in CNT@ Li_2_FeSiO_4_@C can be calculated by the linear relationship between the peak current Ip(A) and the square root of the scan rate *v*^1/2^(*v*^1/2^ *s*^−1/2^) from the CVs (Fig. 5b). Through the equation below [33, 34],

$$ \mathrm{Ip}=2.69\times {10}^5{n}^{3/2}{\mathrm{AD}}_{\mathrm{Li}}^{1/2}{\mathrm{C}}_{\mathrm{Li}}^{\ast }{v}^{1/2} $$

Here, *n* is the number of electrons participating in the reaction, *A* refers to the electrode area, and C^∗^_Li_ stands for the volume concentration of Li^+^ in the electrode. Figure 5b illustrates a good linear relationship between Ip and *v*^1/2^. During the anodic and cathodic reactions, the diffusion coefficients of lithium ions are 7.32 × 10^−11^ and 0.64 × 10^−12^ cm^2^ s^−1^, and these coefficients are superior to previous experimental results [35, 36]. This advantage can be attributed to the excellent electrical conductivity and ion transport efficiency of CNT@Li_2_FeSiO_4_@C during charge and discharge. Electrochemical impedance spectroscopy (EIS) was used to investigate the electrochemical performance of CNT@Li_2_FeSiO_4_@C and CNT@Li_2_FeSiO_4_. Additional file 1: Figure S4 shows the Nyquist plots of the CNT@Li_2_FeSiO_4_@C and CNT@ Li_2_FeSiO_4_ electrode. The Nyquist plots are composed of high-frequency semicircle associated with lithium-ion migration resistance (*R*_SEI_) through the solid electrolyte interface (SEI), intermediate frequency semicircle caused by cathode electrolyte interface charge transfer resistance (*R*_ct_), and low-frequency region tilt line corresponding to the lithium-ion diffusion in the cathode (a Warburg region) [36]. The charge transfer resistance (76.5 Ω) of the CNT@Li_2_FeSiO_4_@C is significantly lower than that of the CNT@Li_2_FeSiO_4_ (193.4 Ω), indicating that the electron transfer speed of CNT@Li_2_FeSiO_4_@C is higher.

## Conclusions

In summary, we have prepared CNT@Li_2_FeSiO_4_@C through a very effective layer-by-layer stacking strategy. The core-shell heterostructure CNT@Li_2_FeSiO_4_@C improves the conductivity, enables rapid extraction/insertion of lithium ions, and relieves the structural damage. As a result, it exhibits high capacity, cycling, and rate performance. Therefore, the CNT@Li_2_FeSiO_4_@C cathode material has a promising prospect in the application of lithium ion battery.

## Additional File

**Additional file 1: Figure S1.** (a) SEM image of CNT, (b) SEM of CNT@SiO_2_. **Figure S2.** Thermogravimetry of CNT@Li_2_FeSiO_4_ and CNT@Li_2_FeSiO_4_@C. **Figure S3.** The dQ/dV vs. voltage plots of CNT@Li2FeSiO4@C at 0.2 C. **Figure S4.** The Nyquist plot of CNT@Li2FeSiO4 and CNT@Li2FeSiO4@C electrode and the equivalent circuit for electrodes.

## Data Availability

All data and materials are fully available without restriction.

## Abbreviations

CNT: Carbon nano-tube
CTAB: Cetyltrimethyl ammonium bromide
CV: Cyclic voltammetric
EDX: Energy-dispersive X-ray spectroscopy
EIS: Electrochemical impedance spectroscopy
HR-TEM: High resolution-transmission electron microscopy
LIBs: Lithium-ion batteries
NMP: *N*-methyl-2-pyrrolidone
PVDF: Polyvinylidene fluoride
SEI: Solid electrolyte interface
SEM: Scanning electron microscopy
TEM: Transmission electron microscopy
TEOS: Tetraethoxysilane
TGA: Thermogravimetric analyzer
XPS: X-ray photoelectron spectroscopy
XRD: X-ray diffraction
