# Supplementary material for: The Core-Shell Heterostructure CNT@Li2FeSiO4@C as a Highly Stable Cathode Material for Lithium-Ion Batteries
Source: Nanoscale Res Lett. 2019 Oct 17;14:326. doi: 10.1186/s11671-019-3165-x (PMC6797695; doi:10.1186/s11671-019-3165-x)
Supplement: Supplementary file 1 — Additional file 1: Figure S1. (a) SEM image of CNT, (b) SEM of CNT@SiO2. Figure S2. Thermogravimetry of CNT@Li2FeSiO4 and CNT@Li2FeSiO4@C. Figure S3. The dQ/dV vs. voltage plots of CNT@Li2FeSiO4@C at 0.2 C. Figure S4. The Nyquist plot of CNT@Li2FeSiO4 and CNT@Li2FeSiO4@C electrode and the equivalent circuit for electrodes. [file 11671_2019_3165_MOESM1_ESM.doc]

**Additional files**

**The core-shell heterostructure CNT@Li_2_FeSiO_4_@C as a highly stable cathode material for lithium-ion batteries**

Tao Peng1,2,[[1]](#footnote-2)*, Wei Guo^1,2^, Yingge Zhang^1,2^,Yangbo Wang^1,2^, Kejia Zhu^1,2^, Yan Guo^1,2^, Yinghui Wang^1,2^, Yang Lu^1,2^, Hailong Yan^1,2^


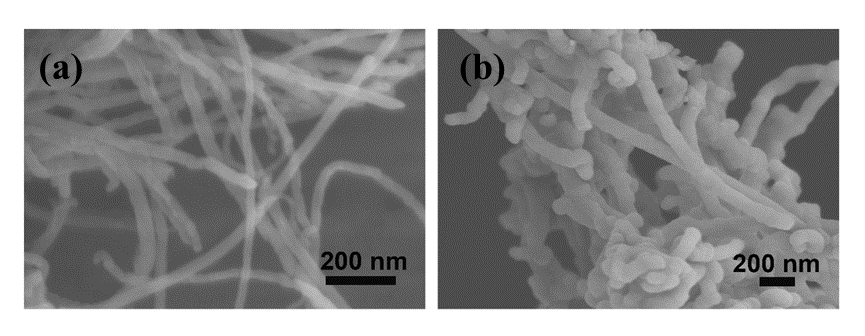


Fig. S1 (a) SEM image of CNT, (b) SEM of CNT@SiO_2_.

Fig. S2 Thermogravimetry of CNT@Li_2_FeSiO_4_ and CNT@Li_2_FeSiO_4_@C.


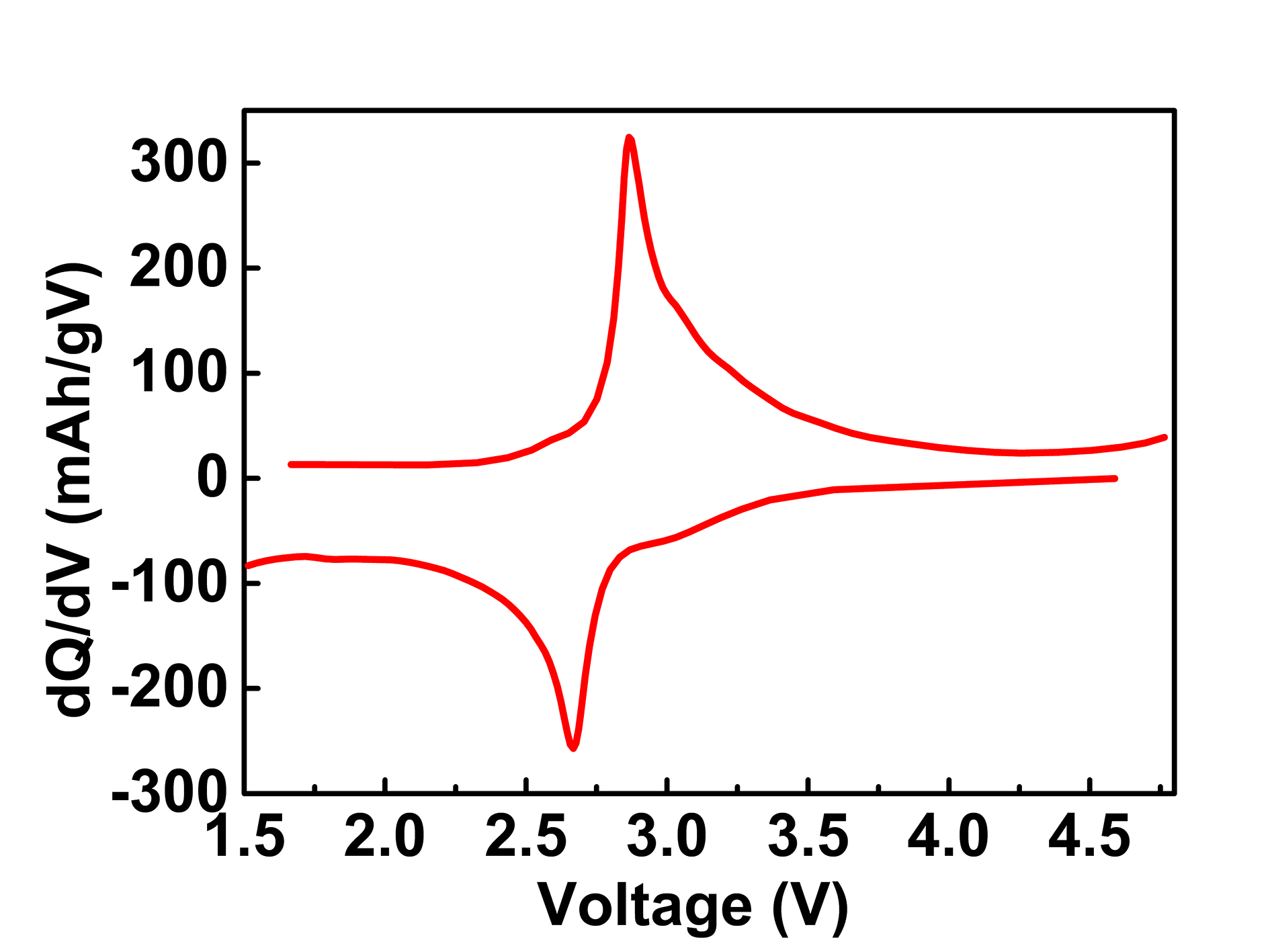


Fig. S3 The dQ/dV vs. voltage plots of CNT@Li_2_FeSiO_4_@C at 0.2 C.


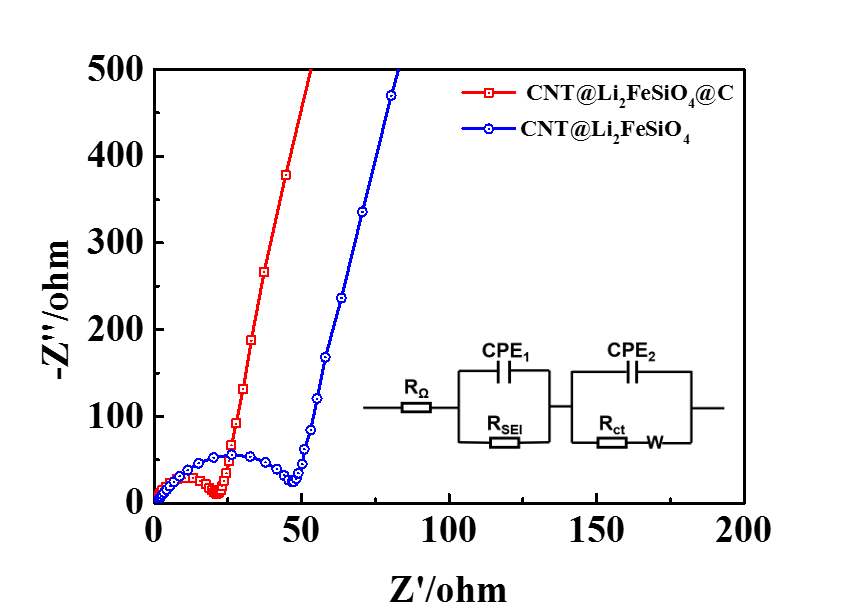


Fig. S4 The Nyquist plot of CNT@Li_2_FeSiO_4_ and CNT@Li_2_FeSiO_4_@C electrode and the equivalent circuit for electrodes.

1. ^^ Corresponding author. E-mail: [tpeng@xynu.edu.cn(T](mailto:tpeng@xynu.edu.cn(T). Peng)

   ^1^ School of Physics and Electronic Engineering, Xinyang Normal University, Xinyang 464000, PR China

   ^2^ Key Laboratory of Microelectronics and Energy of Henan Province, Xinyang Normal University, Xinyang 464000, PR China [↑](#footnote-ref-2)
